# Supplementary material for: Understanding the lithium–sulfur battery redox reactions via operando confocal Raman microscopy
Source: Nat Commun. 2022 Aug 16;13:4811. doi: 10.1038/s41467-022-32139-w (PMC9381601; doi:10.1038/s41467-022-32139-w)
Supplement: Supplementary file 1 — Supplementary Information [file 41467_2022_32139_MOESM1_ESM.pdf]

# Supplementary Information

## Understanding the lithium-sulfur battery redox reactions *via operando* confocal Raman microscopy

Shuangyan Lang[1], Seung-Ho Yu[2], Xinran Feng[1], Mihail R. Krumov[1], and Héctor D. Abruña[1]*

[1]Department of Chemistry and Chemical Biology, Cornell University, Ithaca, New York 14853-1301, USA

[2]Department of Chemical and Biological Engineering, Korea University, Seoul 02841, Republic of Korea

*Corresponding author: Abruña, Héctor D. (hda1@cornell.edu)

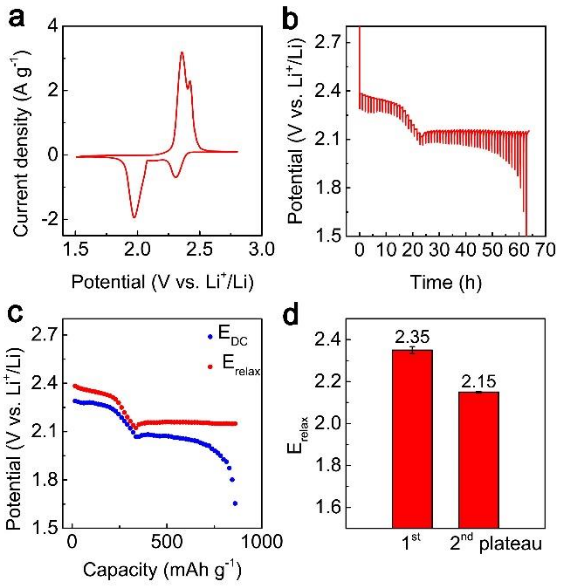


**Supplementary Figure 1.** (**a**) Representative CV of the first cycle of a Li–S coin cell in 1.0 M LiTFSI in DOL/DME electrolyte at a sweep rate of 0.1 mV $s^{-1}$ at 25 ± 1$^{o}$C. (**b**) Raw GITT data during the first discharge process using a cathode of 80 wt% sulfur on carbon paper. (**c**) Processed GITT data. The voltages at the end of each discharge step and relaxation step were extracted from the raw data, marked as $E_{DC}$ and $E_{relax}$ with blue and red dots, respectively. $E_{DC}$ reflects the typical discharge profile, and $E_{relax}$ represents the equilibrium voltages during discharge process[1]. (**d**) Values of $E_{relax}$ for the upper and lower plateaus. The average values of $E_{relax}$ were 2.35 $\pm$ 0.02 and 2.15 $\pm$ 0.01 V. The error bars represent the standard errors of the calculations of average values.

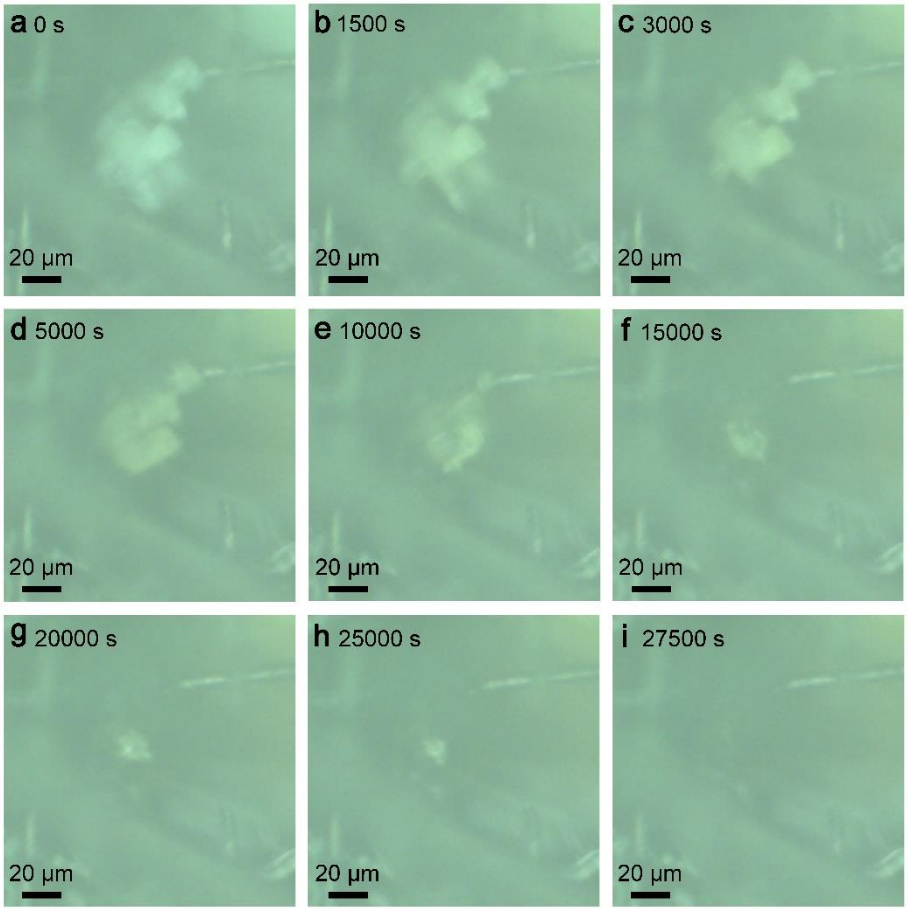


**Supplementary Figure 2.** (**a**-**i**) *Operando* optical images that show the reduction of sulfur clusters at 2.30 V. The surface of sulfur clusters turned yellow during the reduction process due to the formation of polysulfides.

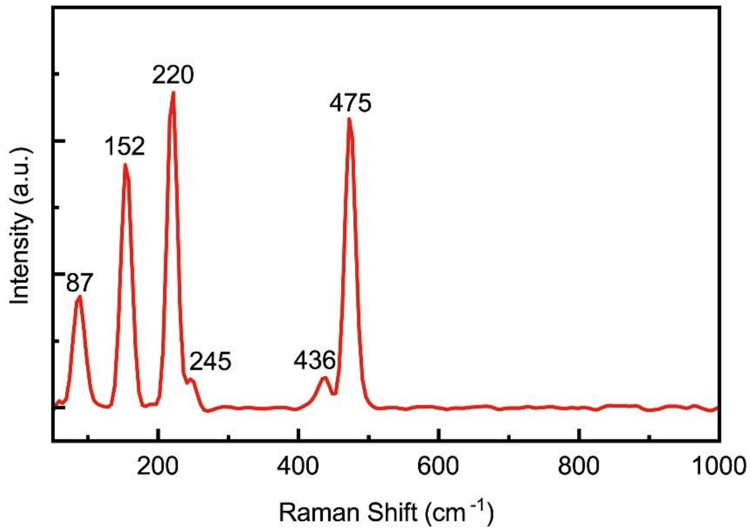


**Supplementary Figure 3.** Full Raman spectrum of a sulfur sample (sulfur: polyvinylidene fluoride =8:2 on the carbon paper). Raman spectrum of the sulfur sample before reduction. The distinctive peaks at 87, 152, 220 and 475 $cm^{-1}$, as well as the smaller ones at 245, 436 $cm^{-1}$ are all assigned to different modes of $S_8$[2, 3]. These peaks are well removed (no overlap) from peaks ascribed to the various polysulfides, making Raman spectroscopy a powerful tool to track the reduction of $S_8$ to the various polysulfides.

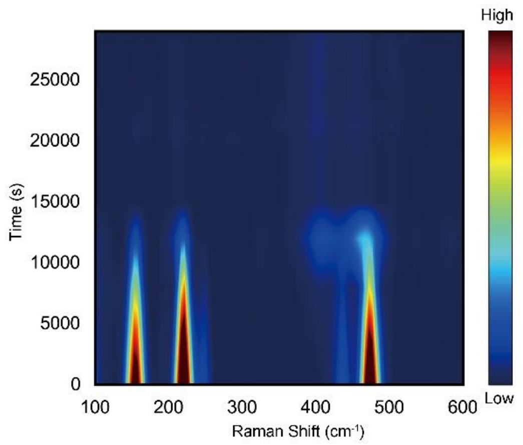


**Supplementary Figure 4.** *Operando* Raman spectra of sulfur reduction at 2.3 V, with the signals of sulfur and polysulfides at the same intensities. The sulfur sample is a slurry of sulfur and polyvinylidene fluoride with the ratio of 8:2 on the carbon paper.

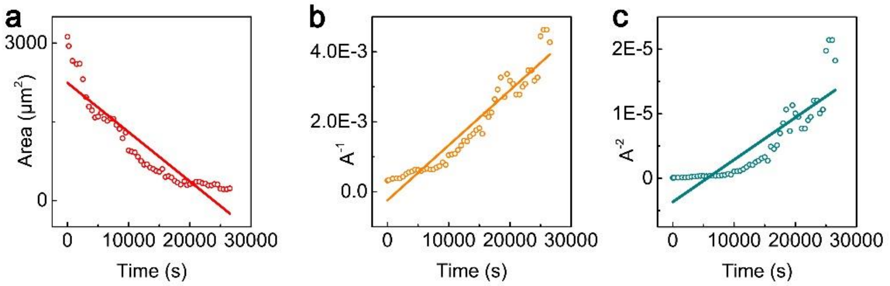


**Supplementary Figure 5. Linear fitting curves of the area change of sulfur clusters with time.** (**a**) Plot of the area vs time, $R^2$=0.849. (**b**) Plot of the reciprocal of area vs time, $R^2$=0.918. (**c**) Plot of the negative quadratic of area vs time, $R^2$=0.765.

The reaction rate dependency of the active surface area of sulfur (A) is expressed by the rate expression (2). According to the expressions of zero, first, second and third-order reactions, A, lnA, $A^{-1}$ and $A^{-2}$ should vary linearly with time, respectively. The corresponding linear fitting curves for sulfur reduction are shown in the inset of Figure 1i and Supplementary Figure 5. The coefficients of determination, $R^2$, were 0.849, 0.979, 0.918 and 0.765 for zero, first, second and third-order models, suggesting a linear relationship between lnA and time.

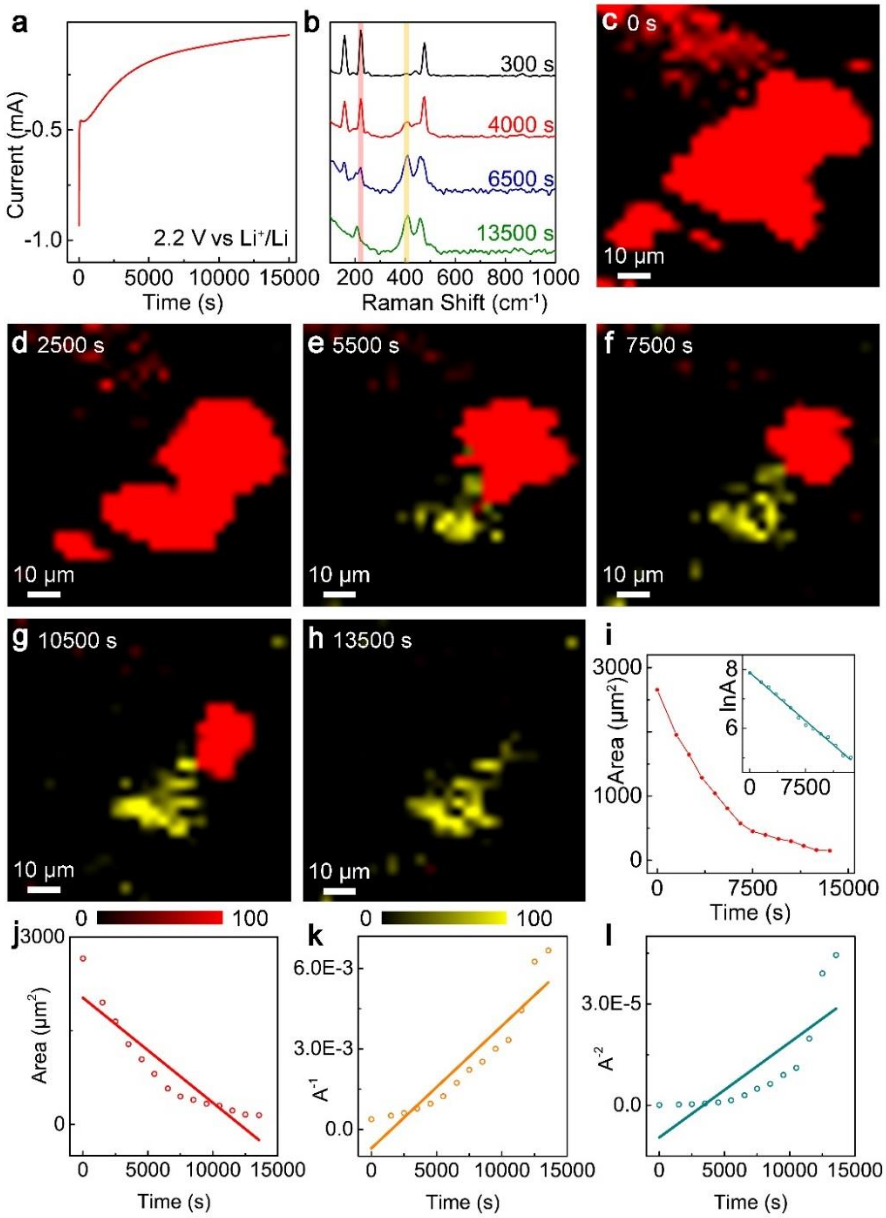


**Supplementary Figure 6. Reduction of sulfur clusters in a Li–S coin cell at 2.20 V vs. $Li^+$/Li at 25 ± 1°C.**

(**a**) Chronoamperometric current-time transient of sulfur reduction at 2.20 V (vs. $Li^+$/Li). *Operando* Raman (**b**) spectra and (**c**-**h**) mapping images upon reduction. The red and yellow colors in (**c**-**h**) represent sulfur and long-chain polysulfides respectively. Color contrasts shown under (**g**) and (**h**), remained consistent in these images for quantification. (**i**) Plot of the area changes of S clusters with time. Inset: Linear fitting of lnA with time, $R^2$=0.993. (**j**-**l**) The linear fitting data of A, $A^{-1}$ and $A^{-2}$ with time, $R^2$ values were 0.846, 0.866 and 0.650 respectively.

Supplementary Figure 6 shows the reduction processes of sulfur clusters at 2.20 V. The evolution of Raman signals was similar to the case at 2.30 V (Supplementary Figures 6b-h). The rate equations of zero, first, second and third-order reactions were further fitted to the changes of sulfur cluster area with time (Supplementary Figures 6i-l). As presented in the inset of Supplementary Figure 6i, the logarithm of area change of sulfur clusters was linear with time,

which is consistent with a first-order kinetics model. The corresponding rate constant was 2.16 × $10^{-4} \pm 0.05 \times 10^{-4}$ $s^{-1}$

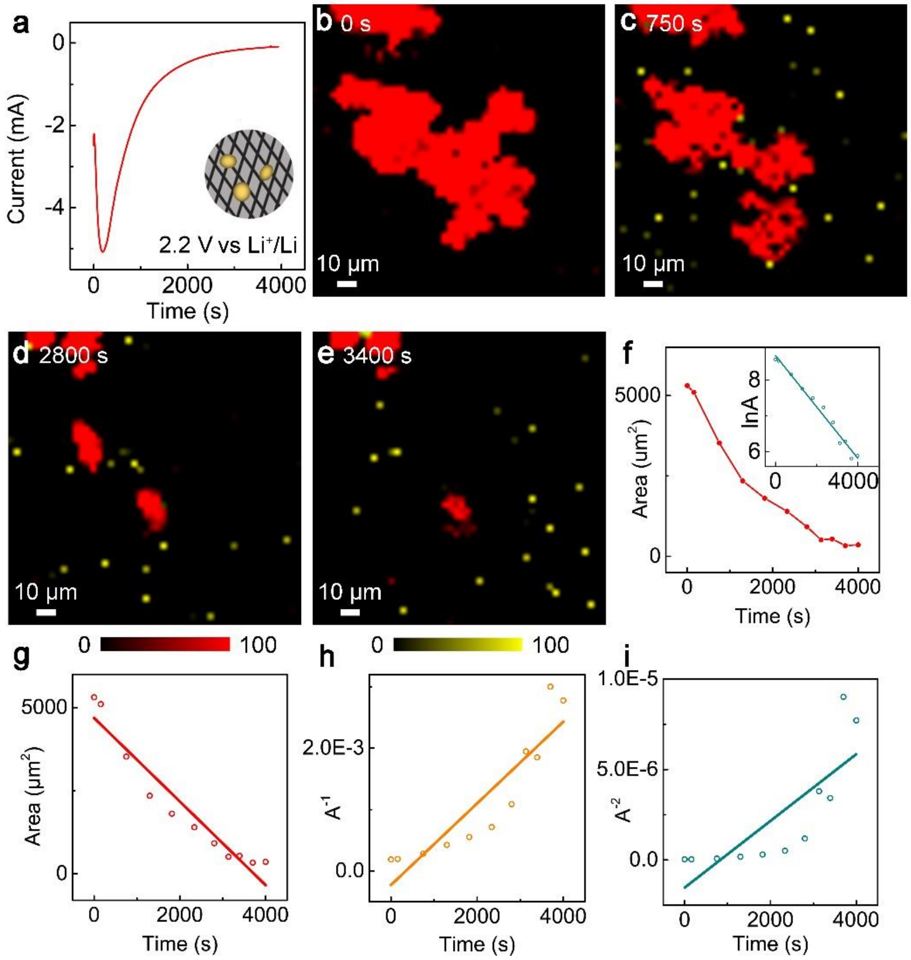


**Supplementary Figure 7. Reduction of a sulfur cathode with a carbon interlayer in a Li–S coincell at 2.20 V vs. $Li^+$/Li at 25 ± 1°C.**

(**a**) Chronoamperometric current-time transient. Inset: schematic of sulfur clusters on the cathode with an additional carbon layer. *Operando* Raman (**b**-**e**) mapping images upon reduction. The red and yellow colors in (**b**-**e**) represent sulfur and long-chain polysulfides respectively. Color contrasts shown under (**d**) and (**e**), remained consistent for quantification. (**f**) Plot of the area changes of S clusters with time. Inset: Linear fitting of lnA with time, $R^2$=0.979. (**g**-**i**) The linear fitting data of A, $A^{-1}$ and $A^{-2}$ with time, $R^2$ values were 0.919, 0.803 and 0.609 respectively.

Supplementary Figure 7 presents the reduction processes at 2.20 V (vs. $Li^+$/Li) of a sulfur cathode with an additional carbon interlayer. The Raman mapping images in Supplementary Figures 7b-e indicate the dissolution of the sulfur clusters as well as the formation and diffusion of long-chain polysulfides. Quantification of the surface area changes with time indicated a first-order relationship, with the $k_S$ of 7.17 × $10^{-4} \pm 0.33 \times 10^{-4}$ $s^{-1}$ (Supplementary Figures 7f-i). It is worth noting here that the rate constant ($k_S$) should not be affected by the contact area. We believe that the addition of the carbon layer accelerates the rate mainly by increasing the contact area of the sulfur clusters with the conductive matrix, rather than the actual surface area of the sulfur clusters

themselves. Thus, the accelerated reaction rate is reflected by an increase in $k_S$ instead of the measured area.

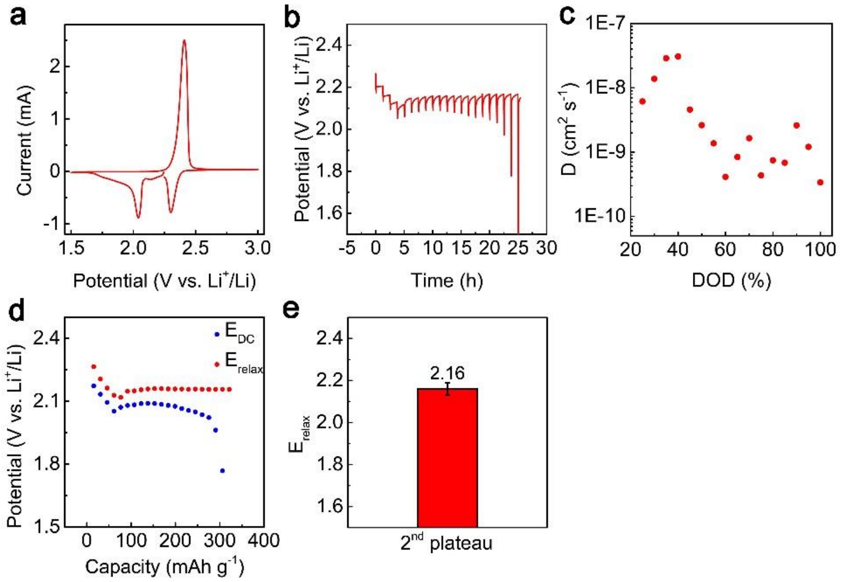


**Supplementary Figure 8.**

(**a**) Representative CV of the first cycle of a Li–polysulfide coin cell using 1 M $Li_2S_4$ as the catholyte at a sweep rate of 0.1 mV $s^{-1}$ at 25 ± 1°C. (**b**) Raw GITT data during the first discharge process. The shallow slope before the plateau could be due to the contribution from the co-existing long-chain polysulfides. (**c**) Diffusion coefficients calculated from GITT. (**d**) Processed GITT data. $E_{DC}$ and $E_{relax}$ after each discharge step and relaxation step were extracted from the raw data, marked as blue and red dots, respectively. (**e**) Value of $E_{relax}$ of the plateau. The average value was 2.16 $\pm$ 0.03 V. The error bar represents the standard error of the calculation of the average value. The 2$^{nd}$ plateau indicates the plateau at about 2.15 V during the discharge process shown in (**b**).

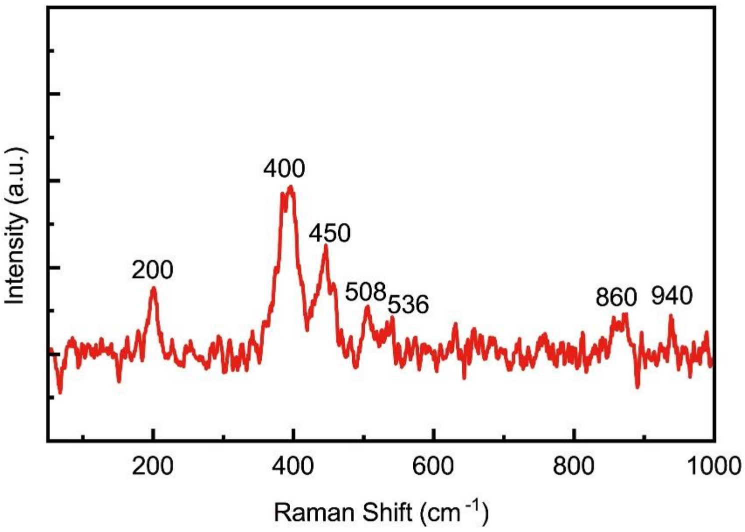


**Supplementary Figure 9.**
Full Raman spectrum of the polysulfide sample. The two peaks at 400 and 450 cm$^{-1}$ were employed to represent the long-chain $Li_2S_x$ (x=6-8) and intermediate $Li_2S_x$ (x=3-5) polysulfides respectively. Additionally, the peaks at 200, 508 and 536 cm$^{-1}$ are also attributed to the polysulfides and have been assigned to $S_4^{2-}$, $S_4^{-}$ and $S_3^{\bullet-}$[2, 4, 5]. Peaks at 860 and 940 cm$^{-1}$ can be attributed to the DOL and DME in the electrolyte[6].

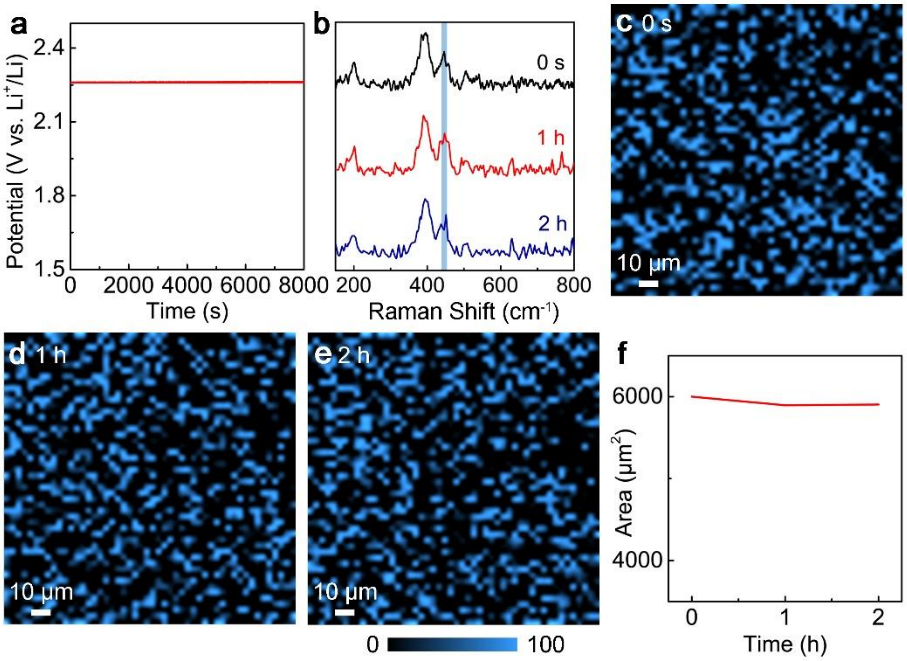


**Supplementary Figure 10.** ***Operando*** **confocal Raman investigation of Li–polysulfide coincells at open circuit at 25 ± 1°C.**
(**a**) Plot of the potential changes at open circuit. *Operando* Raman (**b**) spectra and (**c**-**e**) mapping images during cell storage. The blue color in (**c**-**e**) shows the signal from intermediate polysulfides, centered at 453 cm$^{-1}$ with a band width of 20 cm$^{-1}$. The color contrast is consistent in images and shown under (**e**). (**f**) Raw data of the area changes of the mapping results during cell storage.

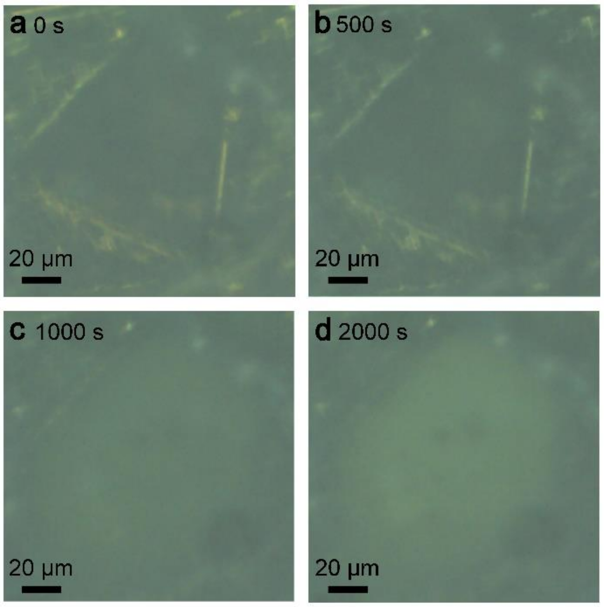


**Supplementary Figure 11.**
(**a**-**d**) *Operando* optical images of the reduction of 1.0 M $Li_2S_4$ catholyte at 2.0 V (vs. $Li^+/Li$). The color intensity decreasing from (**a**) to (**d**) is due to the decrease of polysulfides during the reduction process.

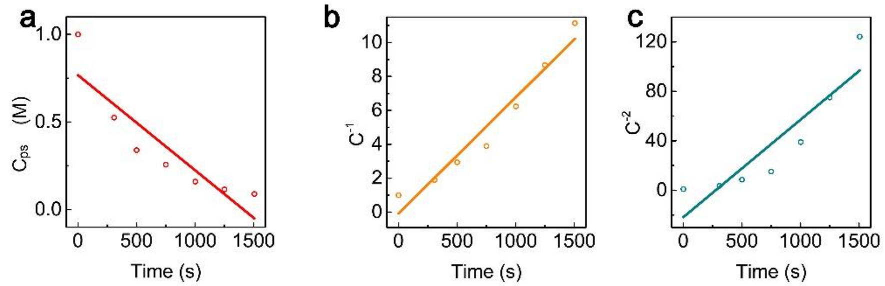


**Supplementary Figure 12.**
(**a**-**c**) Linear fitting of data of the polysulfide concentration (1.0 M $Li_2S_4$ electrolyte), $C^{-1}$ and $C^{-2}$ with time during the reduction at 2.0 V. $R^2$ values were 0.770, 0.944 and 0.796 respectively.
The reaction rate dependency of the concentration (C) is expressed by the rate expression (3). For the 1.0 M $Li_2S_4$ catholyte, the linear fitting curves for different kinetic models are shown in Figure 2f and Supplementary Figure 12. The coefficients of determination, $R^2$, were 0.770, 0.982, 0.944 and 0.796 for zero, first, second and third-order models, suggesting a relatively linear (first-order) relationship between lnC and time (the rate of reduction), yielding $k_{ps}$ ($1.60 \times 10^{-3} \pm 0.09 \times 10^{-3}$ $s^{-1}$) from the slope.

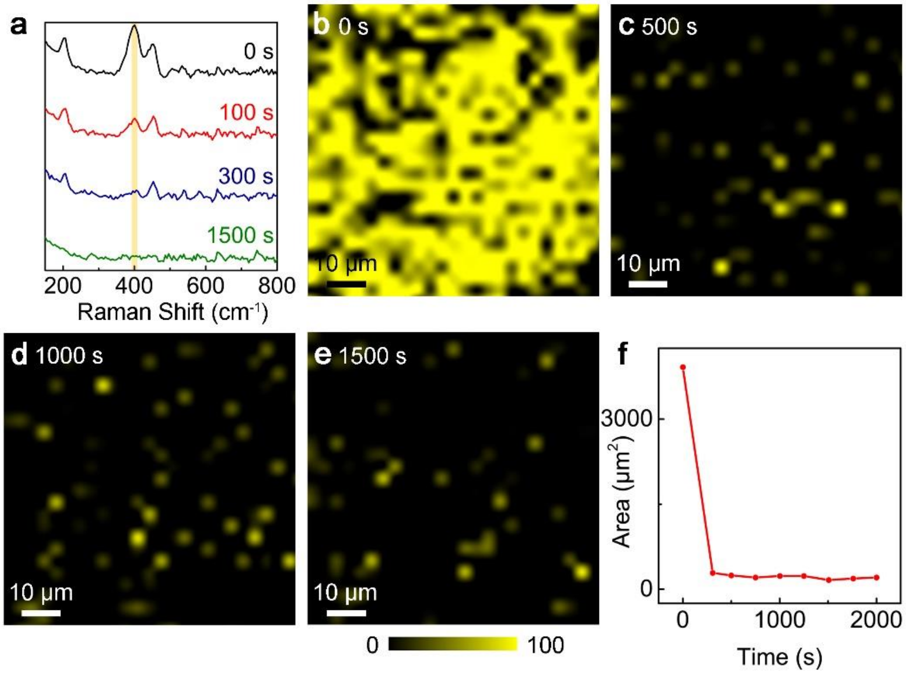


**Supplementary Figure 13. Reduction of long-chain polysulfides at 2.0 V vs. $Li^{+}$/Li.**
*Operando* Raman (**a**) spectra and (**b**-**e**) mapping images during the reduction process. The yellow color in (**b**-**e**) shows the signal from long-chain $S_x^{2-}$, x=6-8 polysulfides, centered at 400 $cm^{-1}$ with a band width of 20 $cm^{-1}$. The color contrast is consistent for quantification, as shown under (**e**). (**f**) Raw data of the area changes of long-chain polysulfides during the reduction process.

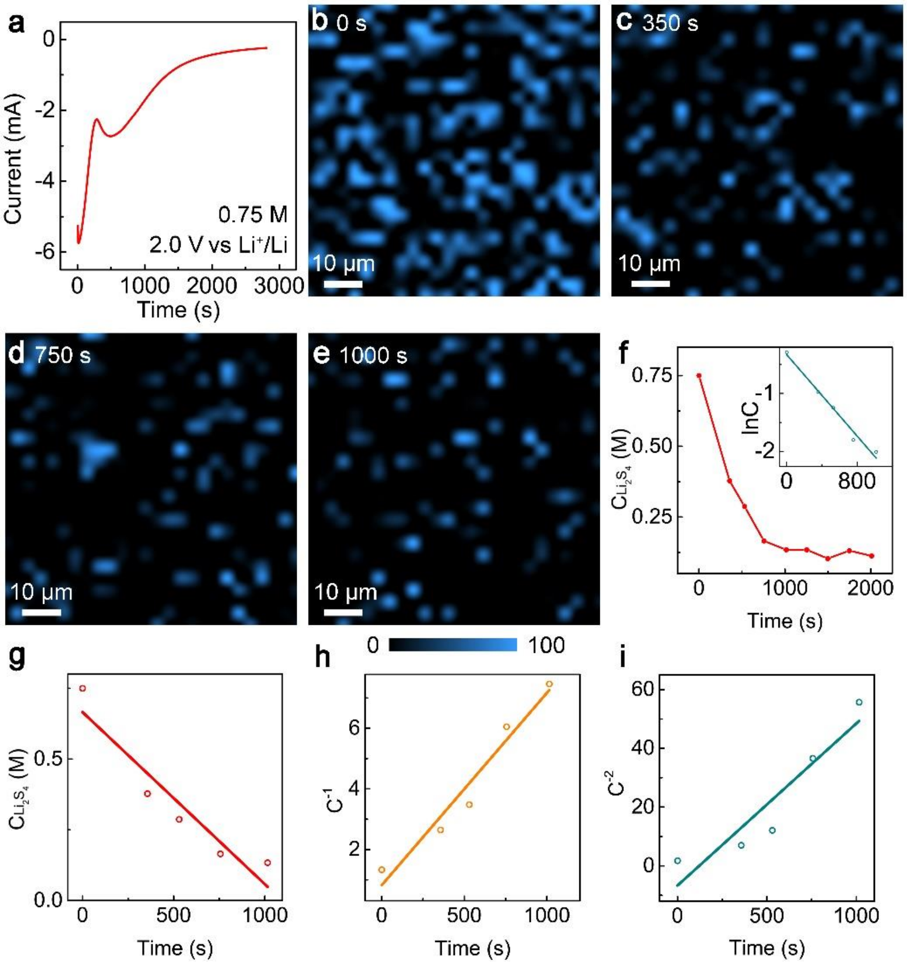


**Supplementary Figure 14. Reduction of polysulfides in Li–polysulfide coincells with 0.75 M $Li_2S_4$ electrolyte at 2.0 V vs. $Li^+$/Li at 25 ± 1°C.**

(**a**) Chronoamperometry current-time transient. *Operando* Raman (**b**-**e**) mapping images upon reduction. The blue color in (**b**-**e**) represents the intermediate polysulfides. Color contrast shown under (**e**), remained consistent among maps for quantification. (**f**) Plot of the change of the concentration with time. Inset: Linear fitting of lnC with time, $R^2$=0.979. (**g**-**i**) The linear fitting data of C, $C^{-1}$ and $C^{-2}$ with time, $R^2$ values were 0.868, 0.938 and 0.834, respectively.

According to equation (3), we employed the zero, first, second and third-order kinetic models to fit the change of the concentration versus time. The results indicated that it followed a first-order reaction law, with the $k_{ps}$ measured to be $1.76 \times 10^{-3} \pm 0.14 \times 10^{-3}$ $s^{-1}$.

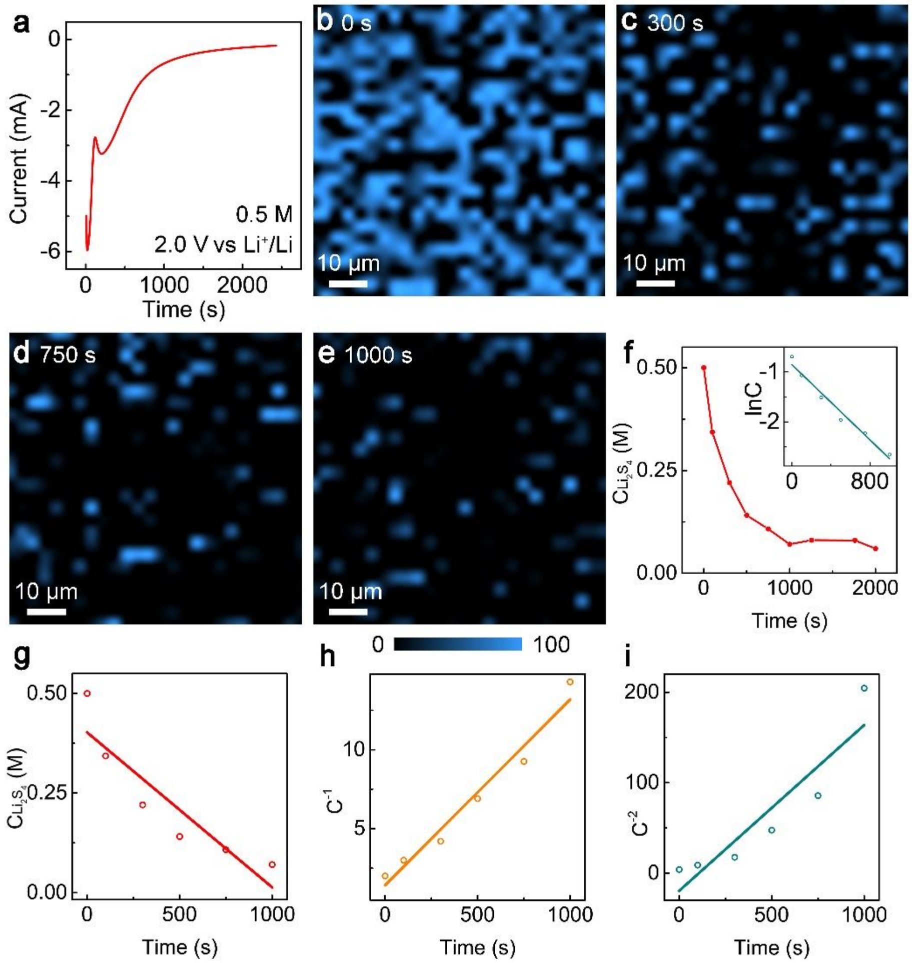


**Supplementary Figure 15. Reduction of polysulfides in Li–polysulfide coincells with 0.5 M $Li_2S_4$ electrolyte at 2.0 V vs. $Li^+$/Li at 25 ± 1°C.**

(**a**) Chronoamperometry current-time transient. *Operando* Raman (**b**-**e**) mapping images upon reduction. The blue color in (**b**-**e**) represents the intermediate polysulfides. Color contrast shown under (**e**), remained consistent among maps for quantification. (**f**) Plot of the change of the concentration with time. Inset: Linear fitting of lnC with time, $R^2$=0.968. (**g**-**i**) The linear fitting data of C, $C^{-1}$ and $C^{-2}$ with time, $R^2$ values were 0.796, 0.959 and 0.871, respectively.

We further fit the change of the concentration versus time in 0.5 M $Li_2S_4$ electrolyte employing the zero, first, second and third-order kinetic models according to equation (3). The results also indicated that it followed a first-order reaction law, with the $k_{ps}$ of $1.88 \times 10^{-3} \pm 0.15 \times 10^{-3}$ $s^{-1}$.

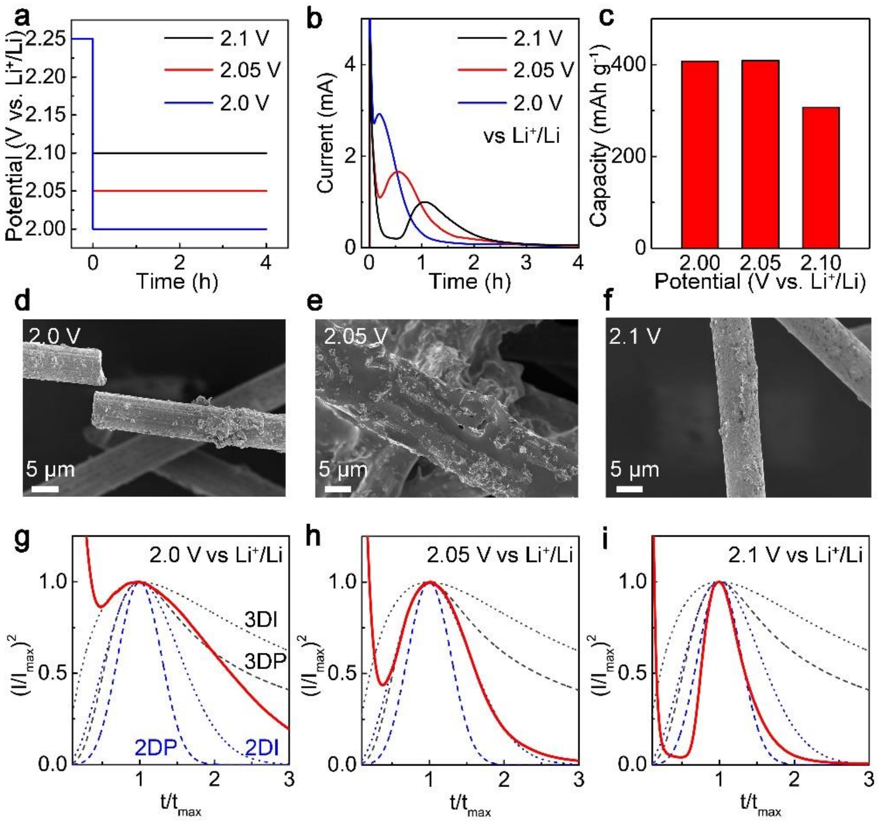


**Supplementary Figure 16. Potentiostatic nucleation and growth of $Li_2S$.** (**a**) Potential step waveforms applied in the chronoamperometric experiments. (**b**) Current-time transients and (**c**) corresponding discharge capacities obtained at different potentials. (**d**-**f**) SEM images of $Li_2S$ deposited on carbon fibers after potentiostatic reactions at different potentials. (**g**-**i**) Dimensionless plots of the current-time transients of $Li_2S$ nucleation and growth with **SH** and **BFT** models.

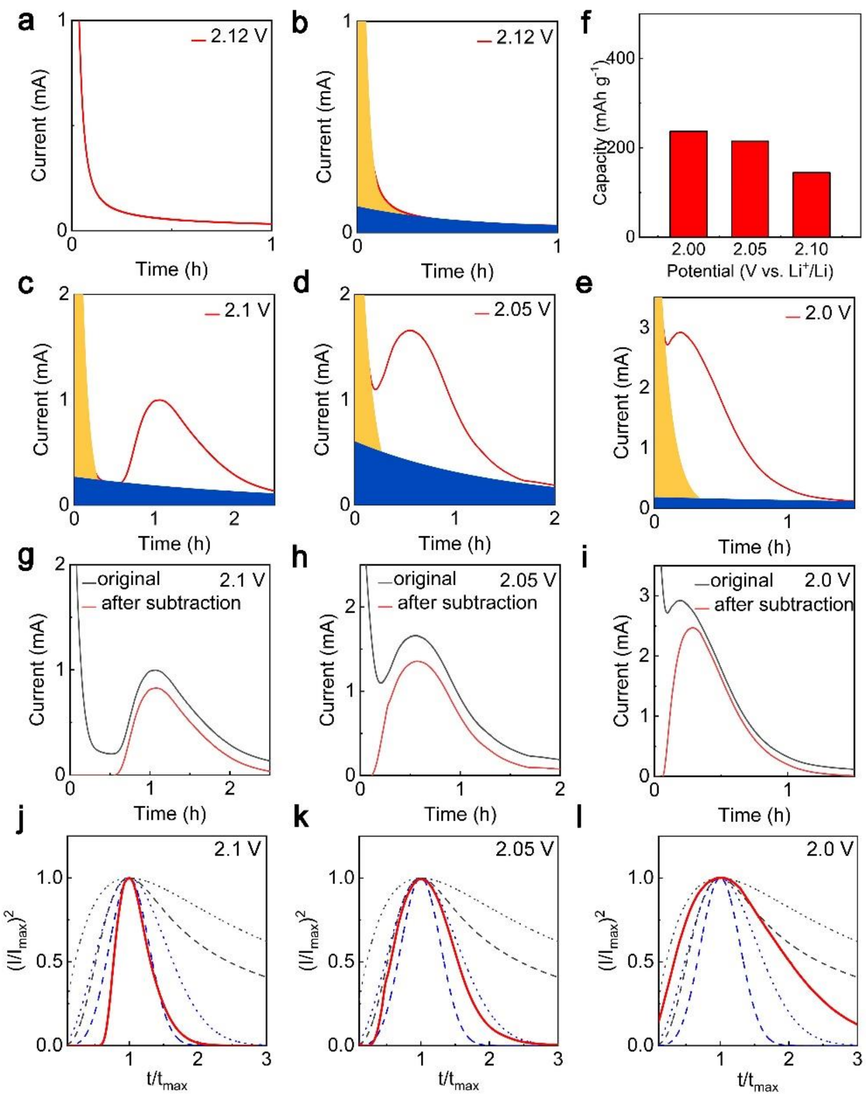


**Supplementary Figure 17. Current-time transients of $Li_2S$ deposition at different potentials (background subtraction).** (**a**) Current-time transients performed at 2.12 V. Background subtraction using two exponential functions at (**b**) 2.12 V, (**c**) 2.1 V, (**d**) 2.05 V and (**e**) 2.0 V (vs. $Li^+/Li$). (**f**) Corresponding discharge capacities after background subtraction. (**g**-**i**) Comparison of the original curves and the ones after background subtraction and (**j**-**l**) the fitting curves at 2.1, 2.05, and 2.0 V.

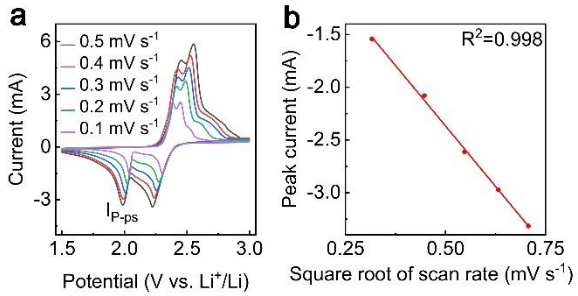


**Supplementary Figure 18. Diffusion-controlled process of polysulfide reduction.** (**a**) CV profiles of a Li–polysulfide battery using $Li_2S_4$ as the catholyte at various scan rates from 0.1 mV $s^{-1}$ to 0.5 mV $s^{-1}$. (**b**) Plot of the polysulfide reduction peak current *vs.* square root of scan rate.

As shown in Supplementary Figure 18b, for the reduction of polysulfides, there is a clear linear relationship between the peak current $I_p$, and the square root of the scan rate $v^{1/2}$. This indicates a diffusion-controlled process according to the Randles–Sevcik equation:

$$I_p = 2.69 \times 10^5 n^{3/2} A D^{1/2} C v^{1/2} \qquad (1)$$

where $I_p$ is the peak current, n is the number of electrons transferred, A is the geometric area of the active electrode, D is the lithium-ion diffusion coefficient, C is the concentration of $Li^+$, and v is the potential scan rate.

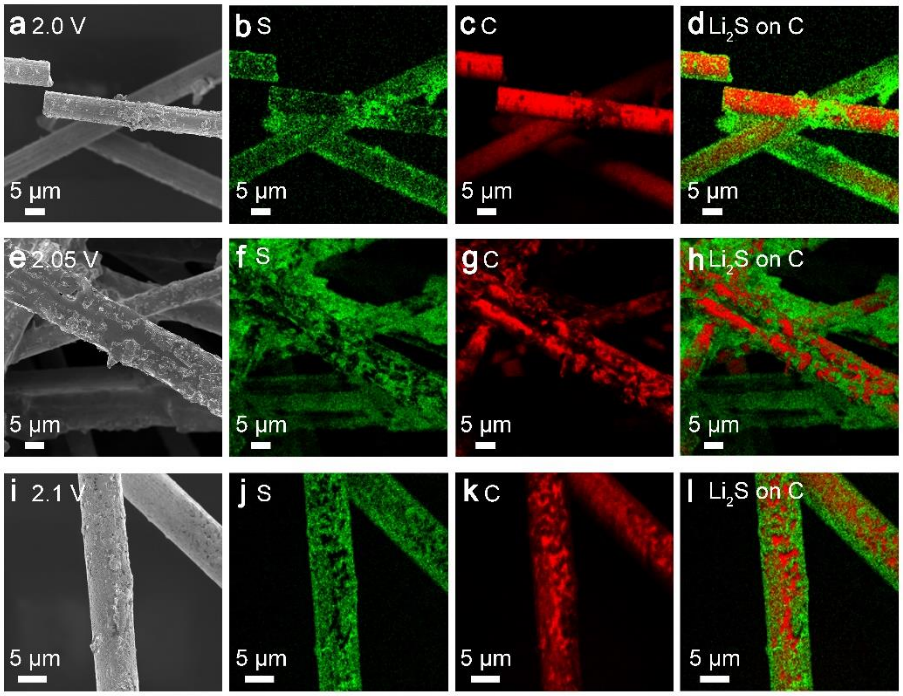


**Supplementary Figure 19.**
SEM images of $Li_2S$ deposited on carbon fibers at different overpotentials, and the corresponding energy-dispersive spectrum (EDS) mapping of elemental S, C and combined images. (**a**-**d**) 2.0 V, (**e**-**h**) 2.05 V, (**i**-**l**) 2.1 V (vs. $Li^+$/Li).

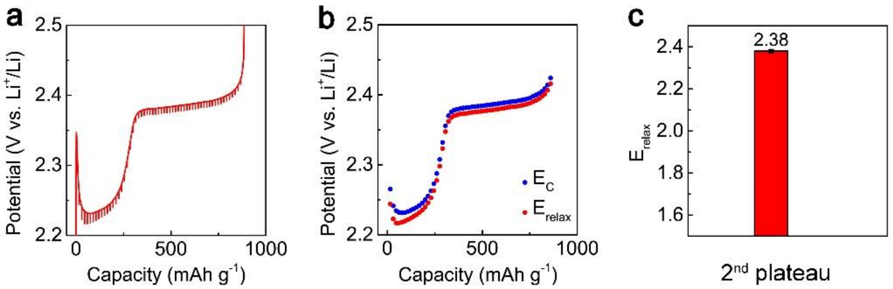


**Supplementary Figure 20.**

(**a**) Raw GITT data during the recharge after first discharge process using a cathode of 80 wt% sulfur on carbon paper. (**b**) Processed GITT data. The voltages at the end of each charge step and relaxation step were extracted from the raw data, marked as $E_C$ and $E_{relax}$ with blue and red dots, respectively. (**c**) The value of $E_{relax}$ for the plateau around 2.4 V. The average value was 2.38 $\pm$ 0.01 V. The error bar represents the standard error of the calculation of the average value. The 2nd plateau indicates the plateau at about 2.38 V during the charge process shown in (**b**).

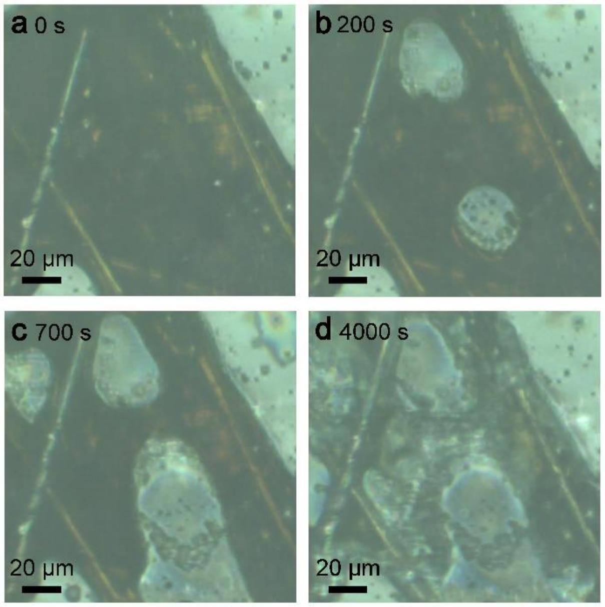


**Supplementary Figure 21.** (**a**-**d**) *Operando* optical images of the oxidation of 1.0 M $Li_2S_4$ catholyte at 2.40 V. The breakage of the homogeneous catholyte, shown by the circles in the center of the images, is related to the phase transformation from soluble polysulfides to solid sulfur.

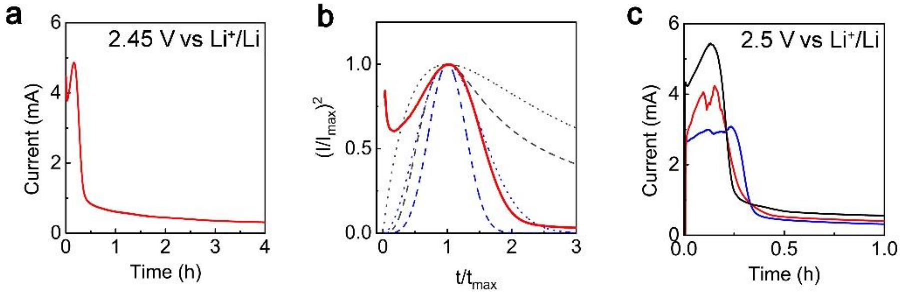


**Supplementary Figure 22. Sulfur nucleation and growth at higher overpotentials.**

(**a**) Current-time transient of sulfur reformation at 2.45 V (vs. $Li^+$/Li). (**b**) Dimensionless fitting of (**a**) with classical BFT and SH models. (**c**) Three trials of sulfur nucleation and growth at 2.50 V (vs. $Li^+$/Li).

As shown in Supplementary Figure 22b, it is clear that the reformation of sulfur at 2.45 V deviates from classical nucleation models. In addition, spilt and multiple peaks can be observed on the current-time transient at 2.50 V. Similar phenomena have been reported previously, when investigating the high-overpotential formation of multiple layers on metal surfaces[7]. We attribute our results to the non-classical sulfur aggregation with larger overpotentials.

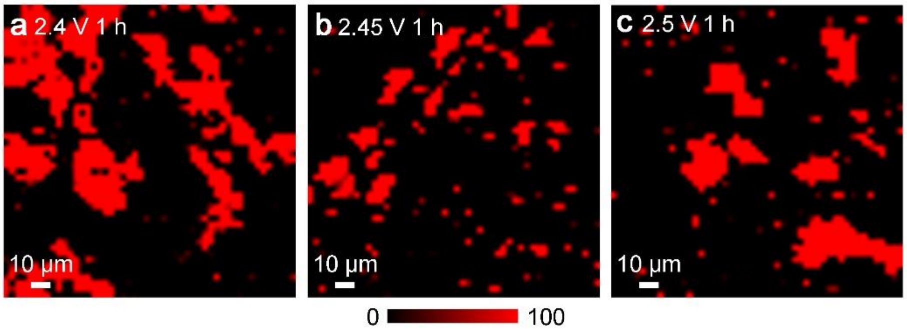


**Supplementary Figure 23.**

Confocal Raman maps of sulfur clusters after 1 h potentiostatic deposition at (**a**) 2.40 V, (**b**) 2.45 V and (**c**) 2.50 V (vs. $Li^+$/Li). The color contrast shown under (**b**), remained consistent for comparison. The red color represents the sulfur clusters, and the black represents the background without sulfur signals.

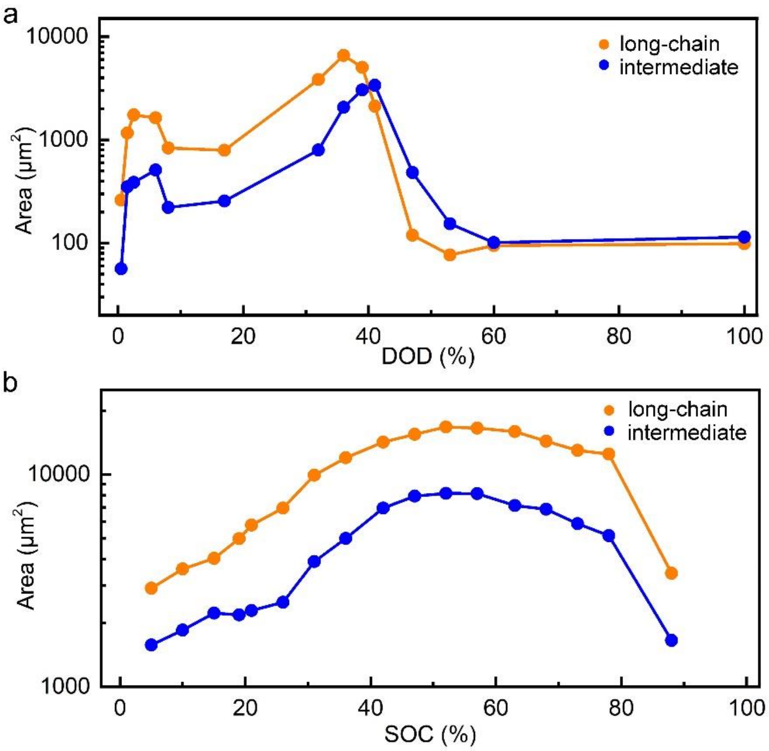

**Supplementary Figure 24.** Area changes of the polysulfides during the (**a**) discharge and (**b**) charge processes in the 150 × 150 μm$^2$ region (window) shown in Supplementary Movie 5.

As shown in Supplementary Figure 24a, at the early stages of the discharge process (0-5% DOD), polysulfides are formed due to the reduction of elemental sulfur. The intensity of the long-chain polysulfides (orange) reaches a localized maximum at around 3% DOD, while the intermediate one (blue) peaks at about 6% DOD. Both of them subsequently decrease due to diffusion processes. The intensities of both long and intermediate length polysulfides increased again at around 30% DOD, which could be related to the further reduction of additional sulfur clusters. We employed the middle region, around 30-50% DOD, to carefully investigate the transformation processes of the polysulfides, as discussed in Figure 4g. In the later stages of discharge, the intensities of the peaks of both long and intermediate length polysulfides decreased significantly and after about 60% DOD there were virtually no further changes in intensity detected, attributed to their further reduction as well as diffusion processes.

It is worth emphasizing that the stepwise reduction and parallel oxidation processes that we proposed are based on the changing trends of different polysulfides as characterized by the relative intensities and their changes. During the discharge processes, non-synchronous changes in the intensities of the long-chain and shorter chain polysulfides were observed, providing clear evidence that the transformations from long-chain are essential to the formation of shorter ones. The prominent presence/function of the intermediate products reveals the characteristics of the stepwise reaction. This is clearly different from their simultaneous changes during the recharge process, directly distinguishing the stepwise discharge mechanism from the parallel recharge one. These phenomena can be due, at least in part, to the slower diffusion processes of the longer chains and the sluggish solid-liquid transformations relative to the liquid-liquid ones, revealing important information of Li-S redox mechanisms and suggesting strategies for effectively catalyzing these processes.

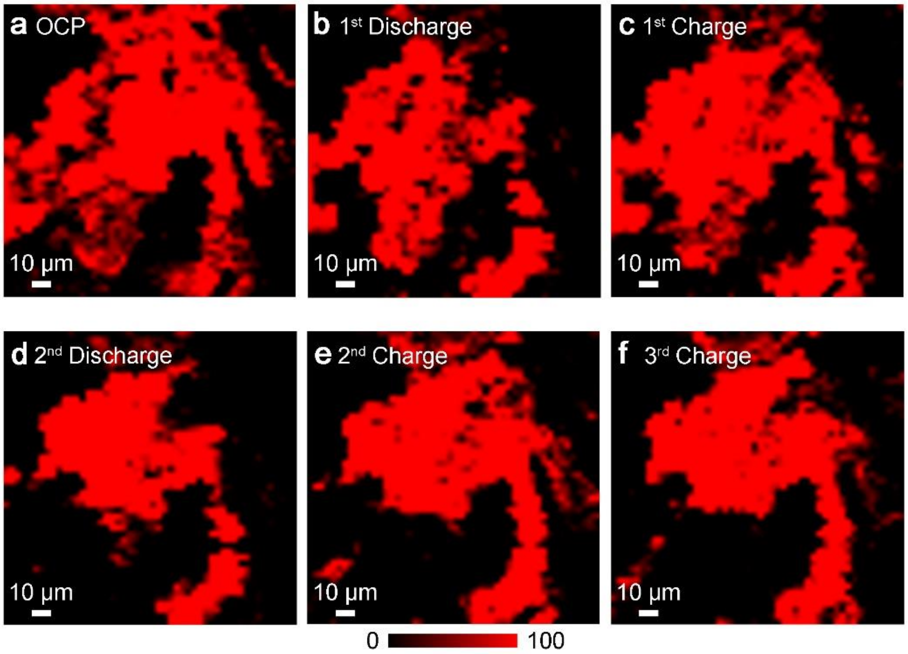


**Supplementary Figure 25.**

(**a**-**f**) *Operando* Raman mapping images showing the evolution of sulfur clusters (with larger sizes) at the cathode during galvanostatic discharge and charge processes at 0.2 C (2.0 mA). The color

contrast shown under (**e**), remained consistent for comparison. The red color represents the sulfur clusters, and the black represents the background without sulfur signals.
As shown in Supplementary Figure 25, the pristine sulfur clusters were not completely dissolved during the initial discharge process. New clusters deposited onto the surrounding areas of the pristine ones during the subsequent charge process. Thus, the inner parts of the pristine sulfur clusters remained electrically disconnected during cycling, causing capacity fade. These results further illustrate the importance of the homogeneous distribution of sulfur clusters on Li–S redox performance.

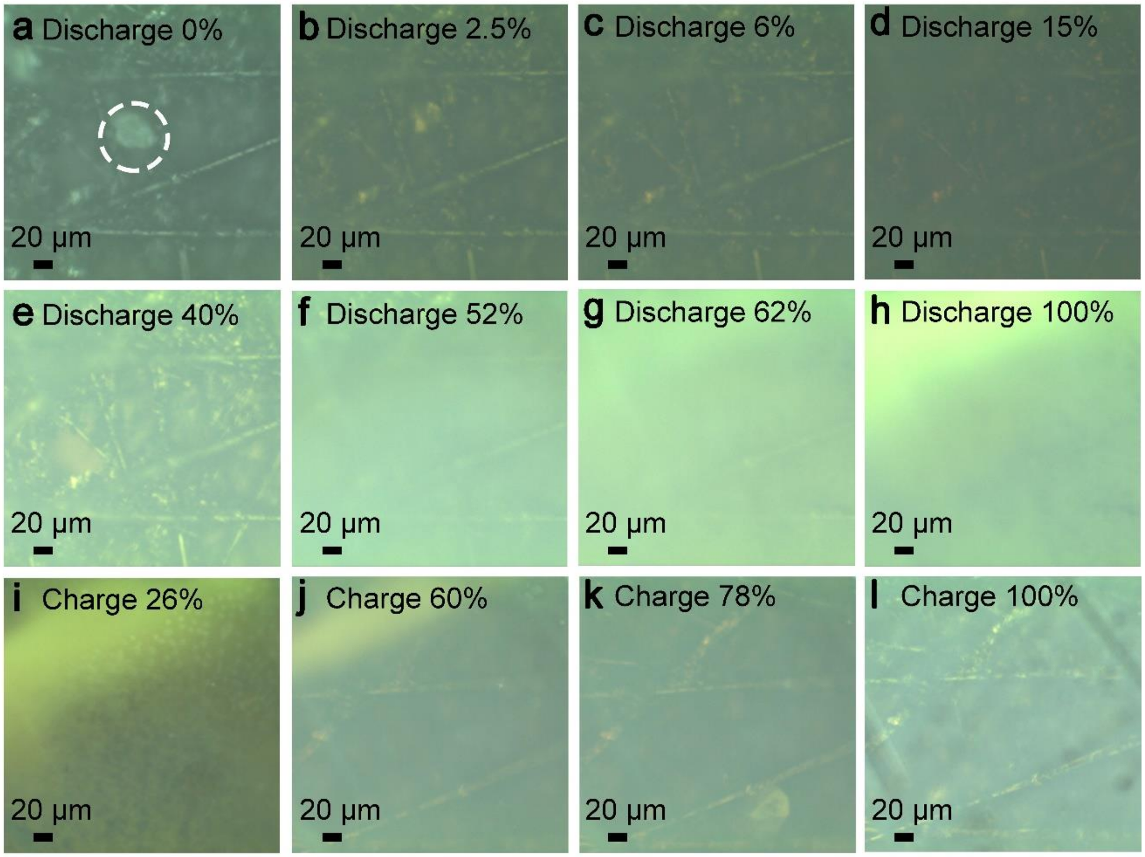


**Supplementary Figure 26.**
*Operando* optical images showing the evolution of sulfur clusters and sulfide species at the cathode during galvanostatic (**a**-**h**) discharge and (**i**-**l**) recharge processes. The brightness of (**e**-**l**) was turned up in an effort to clearly observe the interfaces.
During discharge process, the pristine sulfur clusters (marked by a circle in Supplementary Figure 26a) decreased and completely disappeared at 6% DOD (Supplementary Figure 26c). Subsequently, the interface turned dark and yellow due to the formation of polysulfides. In Supplementary Figure 26f, the entire interface turned blurry which could be due to, at least in part, the formation of insoluble $Li_2S$. During the recharge process, insoluble $Li_2S$ was oxidized back to polysulfides. The evident accumulation decreased and gradually disappeared as illustrated in Supplementary Figures 26i-k. In Supplementary Figure 26l, the interface turned bright along with the further reaction and diffusion of polysulfides.

**Supplementary Table 1. Summary of the state-of-the-art fundamental investigation for Li-S redox mechanisms (non-aqueous Li-S battery systems) using *in situ*/*operando* measurements.** The superscript numbers in the "Year" column refer to the corresponding articles mentioned in the supplementary reference list.

| Year | Electrochemical system | Li–S reduction processes proposed | Main conclusions |
|---|---|---|---|
| 2012[2] | S\|1 M LiTFSI in TEGDME, DOL or their mixture\| Li | $S_8 \rightarrow S_x^{2-}$ (x=4-8) $\rightarrow S_3^{\bullet-}$ | First-time Raman characterization of polysulfides at various discharge and charge states |
| 2013[6] | S\|0.7 M LiTFSI, 0.25 M $LiNO_3$ in DOL/DME\| Li | $S_8 \rightarrow S_x^{2-}$ (x=6-8) $\rightarrow S_x^{2-}$ (x=3-5) | 1. The existence of various polysulfides being dependent on the state of charge<br>2. Solvents have great impact on the Raman lines and the polysulfide creation. |
| 2015[5] | S\|1 M LiTFSI in DOL/TEGDME\| Li | $S_8 \rightarrow S_4^{2-}$, $S_4^-$, $S_3^{\bullet-}$ and $S_2O_4^{2-}$ | $S_8$ is directly reduced to $S_3^{\bullet-}$, on the basis of their similar rate constants under constant potential control, though this is not in agreement with the commonly-accepted reaction pathways of the polysulfides. |
| 2015[8] | S/super P-N composite\|0.5 M $LiCF_3SO_3$, 0.5 M $LiNO_3$ in DOL/DME\| Li | $S_8 \rightarrow S_6^{2-} \rightarrow S_4^{2-}$ and $S_2^{2-}$ | $S_8$ can be detected after the recharging process with the use of the N-doped carbon, revealing that it facilitates the further oxidation of the polysulfides to $S_8$. |
| 2017[9] | S\|1 M LiTFSI in DOL/DME or 0.5 M LiTFSI in $PY_{13}$-FSI\| Li | $S_8 \rightarrow S_8^{n-} \rightarrow S_4^{2-}$, $S_2^{2-}$ and $S^{2-}$ | 1. Similar reaction pathways of the polysulfides in these two electrolytes<br>2. The Raman characterization at Li metal anode shows that the diffusion processes of the polysulfides are slower in ionic liquids. |
| 2020[10] | S\|1 M LiTFSI, 2wt% $LiNO_3$, 10wt% $PES_n$ in DOL/DME\| Li | $S_8 \rightarrow S_{6\text{-}8}^{2-} \rightarrow S_{2\text{-}4}^{2-}$ | Long-chain polysulfides can be observed earlier during the discharge process with the $PES_n$ additive, showing its accelerating effects on the reduction of $S_8$ to the polysulfides. |

**Supplementary Table 2. Main sub-steps of the sulfur reduction process and accompanying disproportionation and conproportionation reactions.**

| Stages | Proposed reactions |
|---|---|
| Solid-liquid reduction of sulfur to polysulfides | $S_8 + 2e^- \rightarrow S_8^{2-}$ |
| Polysulfide transformation | $S_8^{2-} + 2e^- \rightarrow 2S_4^{2-}$ |
| | $S_6^{2-} + e^- \rightarrow 3/2S_4^{2-}$ |
| | $S_7^{2-} + 2e^- \rightarrow S_4^{2-} + S_3^{2-}$ |
| | $S_5^{2-} + 2e^- \rightarrow S_3^{2-} + S_2^{2-}$ |
| Formation of short-chain sulfides | $S_4^{2-} + 2e^- \rightarrow 2S_2^{2-}$ |
| | $S_3^{2-} + 2e^- \rightarrow S^{2-} + S_2^{2-}$ |
| | $S_2^{2-} + 2e^- \rightarrow 2S^{2-}$ |

| | |
|---|---|
| Disproportionation and conproportionation reactions | $S_8^{2-} \rightarrow 1/4S_8 + S_6^{2-}$<br>$S_6^{2-} \rightarrow 2S_3^{\bullet-}$<br>$2S_6^{2-} \rightarrow S_5^{2-} + S_7^{2-}$<br>$S_4^{2-} \rightarrow 3/5S_6^{2-} + 2/5S^{2-}$<br>$2S_4^{2-} \rightarrow S_5^{2-} + S_3^{2-}$<br>$2S_3^{2-} \rightarrow S^{2-} + S_5^{2-}$<br>$3/5S_6^{2-} + 1/5S_2^{2-} \rightarrow S_4^{2-}$ |

**Supplementary Table 3 Expressions of Bewick–Fleischmann–Thirsk (BFT)[11] and Scharifker–Hills (SH)[12] models.**

zF (C mol$^{-1}$) is the molar charge, F=96485 C mol$^{-1}$ is the Faraday constant, k is the growth rate constant (mol cm$^{-2}$ s$^{-1}$), h (cm) is the layer thickness, $N_0$ (cm$^{-2}$) is the number density of isolated centers, M (g mol$^{-1}$) is the molecular weight, and ρ (g cm$^{-3}$) is the density of the deposited material, A (s$^{-1}$) is the nucleation rate constant, $N_\infty$ is the number density of active sites, c (mol cm$^{-3}$) is the molar concentration, and $D_0$ (cm$^2$ s$^{-1}$) is the effective diffusion coefficient.

| Models | Expressions |
|---|---|
| BFT instantaneous nucleation | $I = \frac{2zF\pi MhN_0k^2t}{\rho}\ exp\left(-\frac{\pi N_0M^2k^2t^2}{\rho^2}\right)$<br>$\frac{I}{I_{max}} = \frac{t}{t_{max}}\ exp\left[-\frac{1}{2}\left(\frac{t^2}{t_{max}^2} - 1\right)\right]$ |
| BFT progressive nucleation | $I = \frac{zF\pi Mhk^2AN_0t^2}{\rho}\ exp\left(-\frac{\pi M^2k^2AN_0t^3}{3\rho^2}\right)$<br>$\frac{I}{I_{max}} = \frac{t^2}{t_{max}^2}\ exp\left[-\frac{2}{3}\left(\frac{t^3}{t_{max}^3} - 1\right)\right]$ |
| SH instantaneous nucleation | $I = \frac{zFD^{1/2}c}{\pi^{1/2}t^{1/2}}[1 - exp(-N\pi kDt)]$<br>$\frac{I^2}{I_{max}^2} = 1.9542\frac{t_{max}}{t}\left[1 - exp\left(-1.2564\frac{t}{t_{max}}\right)\right]^2$ |
| SH progressive nucleation | $I = \frac{zFD^{1/2}c}{\pi^{1/2}t^{1/2}}[1 - exp(-AN_\infty\pi k'Dt^2/2)]$<br>$\frac{I^2}{I_{max}^2} = 1.2254\frac{t_{max}}{t}\left[1 - exp\left(-2.3367\frac{t^2}{t_{max}^2}\right)\right]^2$ |

The dimensionless expressions reveal that the detailed interpretation of the curve is determined by the time dependence of the nucleation and growth rates, and by the geometry of the growing phase. The wide application of the classical theories greatly facilities the in-depth investigations of various nucleation and growth mechanisms.

**Supplementary Note 1.**

Given that the open-circuit voltage of Li–$Li_2S_4$ cells is around 2.25 V, three potentials were selected for potentiostatic reduction studies, 2.1, 2.05, and 2.0 V (vs. $Li^+$/Li) (Supplementary Figure 16a).

The current-time transients obtained at the different potentials are shown in Supplementary Figure 16b. In all three cases, the current-time transients exhibited an initial high current and decrease prior to the emergence of a peak, which could be due to relaxation processes along with the reduction of higher-order polysulfides. The peak current, $I_{max}$, is inversely related to the time required to reach that value ($t_{max}$) and increases with larger overpotentials (driving force), indicating higher nucleation rates. The corresponding discharge capacities were calculated as shown in Supplementary Figure 16c. The values of the capacities were 407.6, 408.9 and 306.7 mAh $g^{-1}$ at 2.0, 2.05 and 2.1 V, respectively. The results, after background subtraction (reduction of long chain polysulfides), are presented in Supplementary Figure 17 as references. We further investigated the morphologies of $Li_2S$ after potentiostatic reduction by scanning electron microscopy (SEM) imaging (Supplementary Figures 16d-e). The morphologies of $Li_2S$ deposits change from films to accumulated islands with increasing overpotentials, which can be related to the higher values of peak current at higher overpotentials which, in turn, leads to the higher capacity values.

Supplementary Figures 18 and 8c indicate a diffusion-controlled process and a relative constant diffusion coefficient during the polysulfide reduction. We employed the Bewick–Fleischmann–Thirsk (BFT)[11] and Scharifker–Hills (SH)[12] models to fit the I-t curves (Supplementary Figures 16g-i). The expressions of the models, with the dimensional and dimensionless forms, are provided in Supplementary Table 3. As shown in Supplementary Figures 16g-i and 17j-l, in all three cases without and with background subtraction, the values of $(I/I_{max})^2$ around the peak increase with increasing overpotentials, showing a trend from 2D to 3D nucleation and growth process.

## References


1. Busche, M.R. et al. Systematical electrochemical study on the parasitic shuttle-effect in lithium-sulfur-cells at different temperatures and different rates. *Journal of Power Sources* **259**, 289-299 (2014).
2. Yeon, J.-T. et al. Raman Spectroscopic and X-ray Diffraction Studies of Sulfur Composite Electrodes during Discharge and Charge. *Journal of The Electrochemical Society* **159**, A1308-A1314 (2012).
3. Blanchard, D. & Slagter, M. In operando Raman and optical study of lithium polysulfides dissolution in lithium–sulfur cells with carrageenan binder. *Journal of Physics: Energy* **3** (2021).
4. Zhang, G., Zhang, Z.-W., Peng, H.-J., Huang, J.-Q. & Zhang, Q. A Toolbox for Lithium-Sulfur Battery Research: Methods and Protocols. *Small Methods* **1** (2017).
5. Wu, H.L., Huff, L.A. & Gewirth, A.A. In situ Raman spectroscopy of sulfur speciation in lithium-sulfur batteries. *ACS Appl Mater Interfaces* **7**, 1709-1719 (2015).
6. Hagen, M. et al. In-Situ Raman Investigation of Polysulfide Formation in Li-S Cells. *Journal of The Electrochemical Society* **160**, A1205-A1214 (2013).
7. Fleischmann M, T.H.R. The growth of thin passivating layers on metallic surfaces. *Journal of Electrochemical Society* **110**, 688-698 (1963).
8. Chen, J.-J. et al. Conductive Lewis Base Matrix to Recover the Missing Link of Li2S8 during the Sulfur Redox Cycle in Li–S Battery. *Chemistry of Materials* **27**, 2048-2055 (2015).
9. Zhu, W. et al. Investigation of the reaction mechanism of lithium sulfur batteries in different electrolyte systems by in situ Raman spectroscopy and in situ X-ray diffraction. *Sustainable Energy & Fuels* **1**, 737-747 (2017).
10. Xie, J. et al. Direct Intermediate Regulation Enabled by Sulfur Containers in Working Lithium-Sulfur Batteries. *Angew Chem Int Ed Engl* **59**, 22150-22155 (2020).

11. Bewick A, F.M., Thirsk H R. Kinetics of the Electrocrystallization of Thin Films of Calomel. *Transactions of the Faraday Society* **58**, 2200-2216 (1962).
12. Scharifker B, H.G. Theoretical and experimental studies of multiple nucleation. *Electrochimica acta* **28**, 879-889 (1983).
