# Supplementary material for: Understanding the lithium–sulfur battery redox reactions via operando confocal Raman microscopy
Source: Nat Commun. 2022 Aug 16;13:4811. doi: 10.1038/s41467-022-32139-w (PMC9381601; doi:10.1038/s41467-022-32139-w)
Supplement: Supplementary file 3 — Description of Additional Supplementary Files [file 41467_2022_32139_MOESM3_ESM.pdf]

Description of Additional Supplementary Files

Supplementary Movie 1. Operando Raman mapping video of the sulfur electrode during reduction at 2.30 V vs. Li+ /Li.

Supplementary Movie 2. Operando Raman mapping video of the sulfur electrode during reduction at 2.20 V vs. Li+ /Li.

Supplementary Movie 3. Operando Raman mapping video of the cathode during reduction in 1.0 M Li2S4 electrolyte at 2.0 V vs. Li+ /Li.

Supplementary Movie 4. Operando Raman mapping video of the sulfur electrode during oxidation at 2.40 V vs. Li+ /Li.

Supplementary Movie 5. Operando Raman mapping video of the sulfur electrode during the initial galvanostatic discharge and charge processes.
