# Supplementary material for: Understanding the lithium–sulfur battery redox reactions via operando confocal Raman microscopy
Source: Nat Commun. 2022 Aug 16;13:4811. doi: 10.1038/s41467-022-32139-w (PMC9381601; doi:10.1038/s41467-022-32139-w)
Supplement: Supplementary file 9 — Reporting Summary [file 41467_2022_32139_MOESM9_ESM.pdf]

nature portfolio

Corresponding author(s): Hector D. Abruña

Last updated by author(s): Jul 15, 2022

Reporting Summary

Nature Portfolio wishes to improve the reproducibility of the work that we publish. This form provides structure for consistency and transparency in reporting. For further information on Nature Portfolio policies, see our Editorial Policies and the Editorial Policy Checklist.

Statistics

For all statistical analyses, confirm that the following items are present in the figure legend, table legend, main text, or Methods section.

n/a	Confirmed	
☐	☒	The exact sample size (*n*) for each experimental group/condition, given as a discrete number and unit of measurement
☐	☒	A statement on whether measurements were taken from distinct samples or whether the same sample was measured repeatedly
☐	☒	The statistical test(s) used AND whether they are one- or two-sided<br>*Only common tests should be described solely by name; describe more complex techniques in the Methods section.*
☐	☒	A description of all covariates tested
☐	☒	A description of any assumptions or corrections, such as tests of normality and adjustment for multiple comparisons
☐	☒	A full description of the statistical parameters including central tendency (e.g. means) or other basic estimates (e.g. regression coefficient) AND variation (e.g. standard deviation) or associated estimates of uncertainty (e.g. confidence intervals)
☒	☐	For null hypothesis testing, the test statistic (e.g. *F*, *t*, *r*) with confidence intervals, effect sizes, degrees of freedom and *P* value noted<br>*Give P values as exact values whenever suitable.*
☒	☐	For Bayesian analysis, information on the choice of priors and Markov chain Monte Carlo settings
☐	☒	For hierarchical and complex designs, identification of the appropriate level for tests and full reporting of outcomes
☐	☒	Estimates of effect sizes (e.g. Cohen's *d*, Pearson's *r*), indicating how they were calculated

Our web collection on statistics for biologists contains articles on many of the points above.

Software and code

Policy information about availability of computer code

Data collection	WITec Raman software Control Five 5.1
Data analysis	ImageJ

For manuscripts utilizing custom algorithms or software that are central to the research but not yet described in published literature, software must be made available to editors and reviewers. We strongly encourage code deposition in a community repository (e.g. GitHub). See the Nature Portfolio guidelines for submitting code & software for further information.

Data

Policy information about availability of data

All manuscripts must include a data availability statement. This statement should provide the following information, where applicable:

- Accession codes, unique identifiers, or web links for publicly available datasets
- A description of any restrictions on data availability
- For clinical datasets or third party data, please ensure that the statement adheres to our policy

The authors declare that all experimental data and relevant analysis of this work are available from the corresponding author (HDA) upon reasonable request.

Field-specific reporting

Ecological, evolutionary & environmental sciences study design

All studies must disclose on these points even when the disclosure is negative.

Study description	We report on an investigation of the reaction kinetics of Li–S redox processes and elucidate the underlying mechanism of polysulfide generation/evolution and sulfur redeposition using operando confocal Raman microscopy. The quantitative analysis of the Raman mapping is based on the changes of the projected areas during the reactions of sulfur clusters and polysulfide solutions, which served as proxies for the active surface areas and corresponding concentrations, respectively.
Research sample	The research samples were sulfur clusters and/or polysulfide catholytes as cathode materials of the Li-S and Li-polysulfide cells, respectively. The samples were selected based on a comprehensive consideration of the time required for Raman mapping and cell discharge/charge rates. The Raman spectra and mapping images were obtained with a WITec Alpha300R confocal Raman microscope.
Sampling strategy	For Li–S cells, sulfur electrodes were prepared by coating a slurry of sulfur:polyvinylidene fluoride (8:2) onto pinhole-modified carbon paper (AvCarb EP40, Fuelcell Store). Li2S4 catholytes, with different concentrations, were used to assemble Li–polysulfide cells by mixing stoichiometric amounts of Li2S and sulfur in 1.0 M LiTFSI in DOL/DME at 60 degrees centigrade overnight. The sample size was extracted and quantified from the areas of Raman mapping by ImageJ. The changes of the areas were then converted into the corresponding active surface areas/concentrations to plot vs time, showing clear correlations between the reaction rate and time.
Data collection	The Raman spectra and mapping images were obtained with a WITec Alpha300R confocal Raman microscope and recorded by Dr. Shuangyan Lang.
Timing and spatial scale	The Raman mapping was captured every 5 mins in a 150 x 150 µm2 region during the discharge/charge processes of Li-S cells, on the basis of a comprehensive consideration of the time required for Raman mapping and cell discharge/charge rates.
Data exclusions	No data were excluded from the analyses.
Reproducibility	For Li–S cells, sulfur electrodes were prepared by coating a slurry of sulfur:polyvinylidene fluoride (8:2) onto pinhole-modified carbon paper (AvCarb EP40, Fuelcell Store). The operando experiments were successfully repeated by the authors.
Randomization	The sulfur clusters were randomly deposited onto the cathode materials. For Li-polysulfide cells, the catholytes were homogeneous solutions.
Blinding	The investigators were not blinded to group allocation during data collection and analysis.

Did the study involve field work? [ ] Yes [x] No

Reporting for specific materials, systems and methods

We require information from authors about some types of materials, experimental systems and methods used in many studies. Here, indicate whether each material, system or method listed is relevant to your study. If you are not sure if a list item applies to your research, read the appropriate section before selecting a response.

Materials & experimental systems

n/a	Involved in the study
[x]	[ ] Antibodies
[x]	[ ] Eukaryotic cell lines
[x]	[ ] Palaeontology and archaeology
[x]	[ ] Animals and other organisms
[x]	[ ] Human research participants
[x]	[ ] Clinical data
[x]	[ ] Dual use research of concern

Methods

n/a	Involved in the study
[x]	[ ] ChIP-seq
[x]	[ ] Flow cytometry
[x]	[ ] MRI-based neuroimaging
